# Clinical and non-contrast computed tomography characteristics and disease development in patients with benign pulmonary subsolid nodules with a solid component ≤ 5 mm

**DOI:** 10.1186/s13244-023-01585-5

**Published:** 2024-01-08

**Authors:** Shun Wu, Xiao Fan, Xian Li, Tian-you Luo, Xing-hua Li, Qi Li

**Affiliations:** 1 Department of Radiology, The First Affiliated Hospital of Chongqing Medical University, No. 1 Youyi Road, Yuzhong District, Chongqing, 400016 China; 2 Department of Radiology, Children’s Hospital of Chongqing Medical University, National Clinical Research Center for Child Health and Disorders, Ministry of Education Key Laboratory of Child Development and Disorders, Yuzhong District, Chongqing, China; 3 Department of Pathology, Chongqing Medical University, Yuzhong District, Chongqing, China

**Keywords:** Computed tomography, Ground-glass opacity, Pulmonary nodules, Subsolid nodules

## Abstract

**Objectives:**

To evaluate the clinical and non-contrast computed tomography (CT) features of patients with benign pulmonary subsolid nodules (SSNs) with a solid component ≤ 5 mm and their development trends via follow-up CT.

**Methods:**

We retrospectively collected 436 data from patients who had SSNs with a solid component ≤ 5 mm, including 69 with absorbable benign SSNs (AB-SSNs), 70 with nonabsorbable benign SSNs (NB-SSNs), and 297 with malignant SSNs (M-SSNs). Models 1, 2, and 3 for distinguishing the different types of SSNs were then developed and validated.

**Results:**

Patients with AB-SSNs were younger and exhibited respiratory symptoms more frequently than those with M-SSNs. The frequency of nodules detected during follow-up CT was in the following order: AB-SSNs > NB-SSNs > M-SSNs. NB-SSNs were smaller than M-SSNs, and ill-defined margins were more frequent in AB-SSNs than in NB-SSNs and M-SSNs. Benign SSNs exhibited irregular shape, target sign, and lower CT values more frequently compared to M-SSNs, whereas the latter demonstrated bubble lucency more commonly compared to the former. Furthermore, AB-SSNs showed more thickened interlobular septa and satellite lesions than M-SSNs and M-SSNs had more pleural retraction than AB-SSNs (all *p* < 0.017). The three models had AUCs ranging 0.748–0.920 and 0.790–0.912 in the training and external validation cohorts, respectively. A follow-up CT showed nodule progression in four benign SSNs.

**Conclusions:**

The three SSN types have different clinical and imaging characteristics, with some benign SSNs progressing to resemble malignancy.

**Critical relevance statement:**

A good understanding of the imaging features and development trends of benign SSNs may help reduce unnecessary follow-up or interventions. This retrospective study explores the CT characteristics of benign SSNs with a solid component ≤ 5 mm by comparing AB-SSNs, NB-SSNs, and M-SSNs and delineates their development trends via follow-up CT.

**Key points:**

1. Different subsolid nodule types exhibit distinct clinical and imaging features.

2. A miniscule number of benign subsolid nodules can progress to resemble malignancy.

3. Knowing the clinical and imaging features and development trends of benign subsolid nodules can improve management.

**Graphical Abstract:**

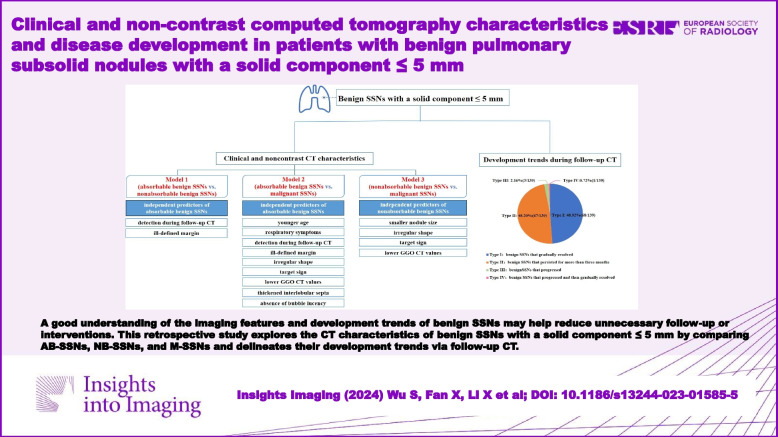

**Supplementary Information:**

The online version contains supplementary material available at 10.1186/s13244-023-01585-5.

## Introduction

The extensive usage of chest computed tomography (CT) for lung cancer screening has led to an increased detection of pulmonary subsolid nodules (SSNs), which can be categorized into pure ground-glass nodules and part-solid nodules. Early-stage lung adenocarcinoma is often detected as SSNs on CT and usually has a good prognosis after surgical resection. Although most SSNs are malignant, some benign diseases can also present with SSNs, thereby resulting in unnecessary follow-up or interventions. Thus, a thorough understanding of the CT features and development trends during follow-ups of benign SSNs can facilitate early diagnosis and enhance management.

Prior studies have shown that sublobectomy is beneficial in patients with lung adenocarcinoma in situ or minimally invasive adenocarcinoma, which usually manifest as SSNs with a solid component ≤ 5 mm on CT [1–7]. Although previous research has investigated the CT differences between benign and malignant SSNs (M-SSNs) [8–14], the non-contrast CT characteristics of absorbable benign SSNs (AB-SSNs), nonabsorbable benign SSNs (NB-SSNs), and M-SSNs with a solid component ≤ 5 mm have not yet been thoroughly investigated. Some scholars have indicated that follow-up CT can show benign SSNs to be resolved or persistent [8–10]. However, in clinical practice, we have found evidence of progression in a small number of benign SSNs on follow-up CT. This mimics malignancy and has been poorly reported.

Therefore, this study aimed to evaluate the clinical and non-contrast CT features of benign SSNs with a solid component ≤ 5 mm by comparing AB-SSNs, NB-SSNs, and M-SSNs and to delineate their development trends via follow-up CT.

## Methods

### Definitions of AB-SSNs, NB-SSNs and M-SSNs

In this study, AB-SSNs refer to SSNs with a solid component ≤ 5 mm that resolved during follow-up CT, NB-SSNs refer to SSNs (that were surgically confirmed as benign) with a solid component ≤ 5 mm that persisted for more than 3 months or progressed without absorption on follow-up CT, and M-SSNs refer to SSNs with a solid component ≤ 5 mm that were surgically confirmed as malignant.

### Patients

**Fig. 1:**
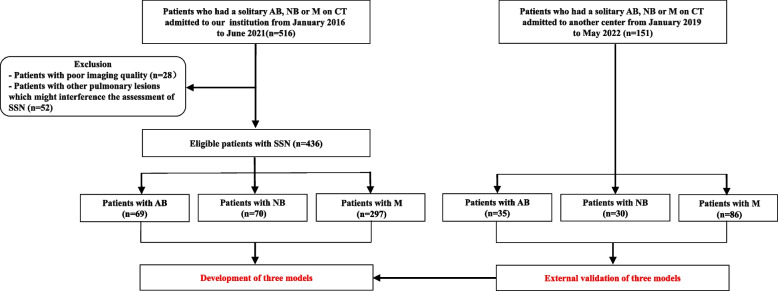
The flow diagram of the study population

The study protocol was approved by the ethics committee of the First Affiliated Hospital of Chongqing Medical University. The need for informed consent was waived due to the retrospective nature of this study. We collected the CT imaging and clinical data of 516 patients with SSNs at our institution between January 2016 and June 2021. The inclusion criteria were as follows: (1) patients who underwent chest non-contrast CT scans; (2) patients who had a solitary AB-SSN, NB-SSN, or M-SSN on CT. Patients were excluded according to the following criteria: (1) poor CT imaging quality due to obvious respiratory motion artifacts; (2) other pulmonary lesions that might interfere with the morphological assessment of SSNs. Finally, 69 patients with AB-SSNs, 70 with NB-SSNs, and 297 with M-SSNs were included in this study. All NB-SSNs were pathologically confirmed as inflammatory nodules. Among patients with M-SSNs, there was pathological confirmation of adenocarcinoma in situ in 98*,* minimally invasive adenocarcinoma in 165, and invasive adenocarcinoma in 34. Relevant clinical data were also collected. An additional 151 patients, including 35 with AB-SSNs, 30 with NB-SSNs, and 86 with M-SSNs admitted to another center between January 2019 and May 2022 and meeting the aforementioned inclusion and exclusion criteria, were included as an external validation cohort. The flow diagram of the study population is shown in Fig. 1.

### CT protocols

Chest CT examinations were performed using one of the following three multidetector CT scanners: Somatom Definition FLASH (Siemens Healthcare), Somatom Perspective (Siemens Healthcare), or Discovery CT750 HD (GE Healthcare). All patients underwent non-contrast CT scans covering the thoracic inlet to the lung base. The following scanning parameters were used: tube voltage, 110–120 kVp; tube current, 50–250 mAs (automatic tube current modulation technology); and scanning slice thickness/interval, 5 mm/5 mm. Thin-section CT images were reconstructed with a slice thickness and interval of 0.625–1.25 mm for the GE scanner and 0.6–1.0 mm for the two Siemens scanners [9].

### CT image analysis

Two radiologists experienced in chest imaging were blinded to patient data and interpreted the CT images independently on a picture archiving and communication system (PACS) workstation (Vue PACS, Carestream). Any disagreements were resolved by discussion until a consensus was reached. The recorded non-contrast CT features of SSNs were as follows [9]:

1. Detected time (either first detected on the initial CT or first detected on the follow-up CT)
2. Nodule location (upper, middle, or lower lung lobe)
3. Size (maximum nodule diameter in the CT lung window setting)
4. Margin (well-defined [clear border] or ill-defined [partially or totally blurred border])
5. Shape (regular [round or oval] or irregular [uneven contour that could not be classified as round or oval])
6. Intranodular characteristics, including density (pure ground-glass opacity [pGGO] or part-solid opacity [PSO]), CT value of the GGO area (the largest slice of the nodule was selected on the axial CT image in the lung window and then, for nodules with pGGO, a circular region of interest [ROI] was drawn covering more than two-thirds of the lesion; for nodules with PSO, the largest possible circular ROI of the GGO was drawn avoiding solid components, vessels, and bronchi. The same ROI was placed on three adjacent images using the copy-and-paste function. Using these images, we recorded the average value [calculated as the CT value of the GGO area]), the presence of a target sign (characterized by a focal, circular, solid component in the center of the SSN, similar to a target), the presence of an air bronchogram (branched or tubular air structures within the lesion), the presence of a bubble lucency (round or oval air attenuation within the lesion), and the presence of an abnormal intranodular vessel sign (vessels in the nodule manifesting as dilatated, distorted, or increased branches)
7. Extra-nodular characteristics, including thickened interlobular septa; satellite lesions (additional lesions ≤ 3 cm from the nodule), pleural attachment (lesions attached to the pleura with the margin obscured by the pleura), and pleural retraction (linear structures connecting the tumor and pleura).

For patients with sequential CT scans, image analyses were based on the earliest CT data of SSNs before treatment.

### Follow-up CT data

The developmental trends of benign SSNs observed on follow-up CT were carefully analyzed. Fifty-three patients had undergone two follow-up CT scans, 11 had undergone three follow-up CT scans, 4 had undergone four follow-up CT scans, and 1 had undergone six follow-up CT scans. The mean observation period was 159 ± 232 days (range, 8–1734 days), and the mean follow-up interval was 125 ± 165 days (range, 6–735 days).

### Statistical analyses

Statistical analyses were performed using SPSS Statistics for Windows, version 19.0 (IBM Corp., Armonk, NY, USA). Interrater agreement on non-contrast CT features was evaluated using the intraclass correlation coefficient (ICC). An ICC value > 0.75 was considered good agreement. For continuous variables, single-sample Kolmogorov–Smirnov analyses were used to test the homogeneity of variance. Normally distributed data were expressed as means ± standard deviations and nonnormally distributed data as medians ± interquartile ranges. Two independent-sample Student’s *t*-tests were used to assess normally distributed variables, and the Mann–Whitney *U* test was used for nonnormally distributed variables. Categorical variables were expressed as numbers and percentages, and chi-squared tests were used to compare variables between groups. Pairwise analyses were used for between-group differences. After Bonferroni correction, a *p*-value of < 0.017 was considered statistically significant. Multivariate logistic regression analyses were performed with clinical and non-contrast CT characteristics that differed significantly between groups to identify independent factors that could be used to diagnose benign SSNs. The final regression models were selected using the forward condition method. The areas under the curve (AUCs) and accuracy were used to evaluate their diagnostic performance. For the regression analyses, a two-tailed *p-*value of < 0.05 was considered statistically significant.

## Results

### Consistency assessments

The consistency assessments between two observers were good for all non-contrast CT features (all *p* < 0.001) (Table 1).

**Table 1:** The consistency assessments between two observers

CT features	ICC value	*p* value
Nodule location	1.000	< 0.001
Size	0.912	< 0.001
Margin	0.879	< 0.001
Shape	0.892	< 0.001
Density	0.934	< 0.001
Target sign	0.934	< 0.001
Air bronchogram	0.943	< 0.001
Bubble lucency	0.954	< 0.001
Abnormal intranodular vessel sign	0.886	< 0.001
Thickened interlobular septa	0.886	< 0.001
Satellite lesions	0.910	< 0.001
Pleural attachment	0.961	< 0.001
Pleural retraction	0.947	< 0.001
CT value of GGO areas	0.930	< 0.001

*CT* Computed tomography, *ICC* Intraclass correlation coefficient, *GGO* Ground glass opacity

### Comparison of clinical characteristics among AB-SSNs, NB-SSNs and M-SSNs

The clinical data of patients with AB-SSNs, NB-SSNs, and M-SSNs are displayed in Table 2. Patients with AB-SSNs were found to be significantly younger and more likely to have respiratory symptoms than those with M-SSNs (*p* < 0.017). The proportion of nodules detected during follow-up CT was highest in AB-SSNs, followed by NB-SSNs and M-SSNs (*p* < 0.017). However, no significant differences were observed between groups in terms of sex or smoking history (all *p* > 0.017).

**Table 2:** Comparison of clinical characteristics among absorbable benign, nonabsorbable benign and malignant subsolid nodules

Characteristics	AB-SSNs (*n* = 69)	NB-SSNs (*n* = 70)	M-SSNs (*n* = 297)	*p*_1_*	*p*_2_*	*p*_3_*
Sex				0.662 ^a^	0.230 ^a^	0.075 ^a^
Male	29 (42.03%)	32 (45.71%)	102 (34.34%)			
Female	40 (57.97%)	38 (54.29%)	195 (65.66%)			
Age (years)				0.055 ^b^	**0.004 **^**c**^	0.879 ^c^
Average	49 ± 14	53 ± 11	54 ± 14			
Range	25–81	16–76	25–82			
Smoking history				0.810 ^a^	0.065 ^a^	0.096 ^a^
Smoker	21 (30.43%)	20 (28.57%)	60 (20.20%)			
Nonsmoker	48 (69.57%)	50 (71.43%)	237 (79.80%)			
Respiratory symptoms				0.662 ^a^	**0.012 **^**a**^	0.056 ^a^
Presence	19 (27.54%)	17 (24.29%)	44 (14.81%)			
Absence	50 (72.46%)	53 (75.71%)	253 (85.19%)			
Detected time				**0.008** ^**a**^	** < 0.001** ^**a**^	** < 0.001** ^**a**^
Initial CT	50 (72.46%)	63 (90%)	297 (100%)			
Follow-up CT	19 (27.54%)	7 (10%)	0 (0%)			

*AB-SSNs* Absorbable benign subsolid nodules, *NB-SSNs* Nonabsorbable benign subsolid nodules, *M-SSNs* Malignant subsolid nodules, *CT* Computed tomography

^*^Bonferroni correction in which *p* < 0.017 is significant. *p*_1_: the *p*-value of AB-SSNs vs. NB-SSNs; *p*_2_: the *p-*value of AB-SSNs vs. M-SSNs; *p*_3_: the *p*-value of NB-SSNs vs. M-SSNs

^a^Chi-squared test

^b^Two independent-sample Student’s *t*-tests

^c^Mann–Whitney *U* test

### Comparison of non-contrast CT features among AB-SSNs, NB-SSNs and M-SSNs

**Fig. 2:**
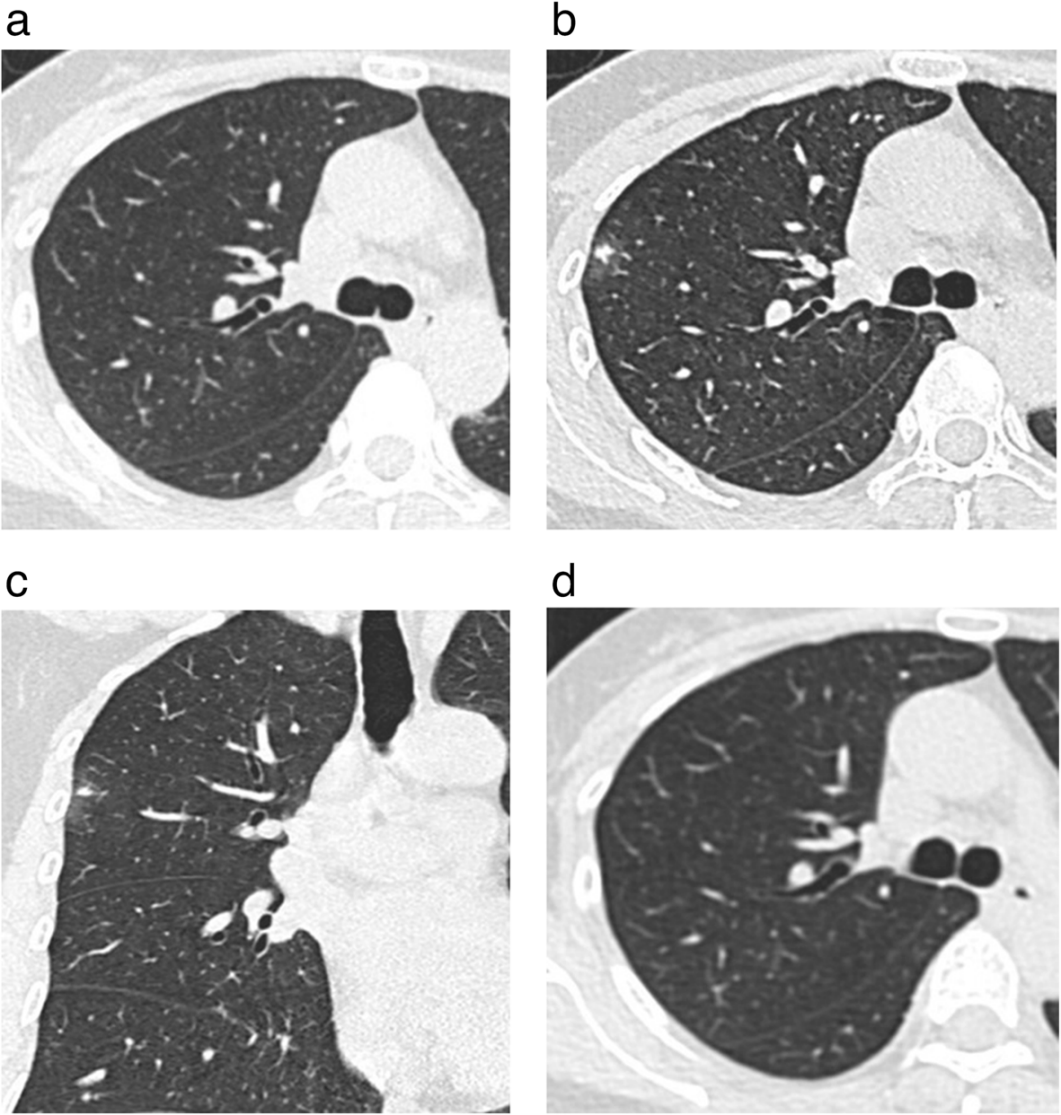
Absorbable benign pulmonary subsolid nodule in a 48-year-old woman. **a** The initial axial lung window CT image showed a normal right upper lobe. **b**, **c** Four months later, the axial and coronal lung window CT images showed a subsolid nodule with an ill-defined margin and a target sign in the right upper lobe. **d** Ten months later, the axial lung window CT image showed that the nodule was resolved

**Fig. 3:**
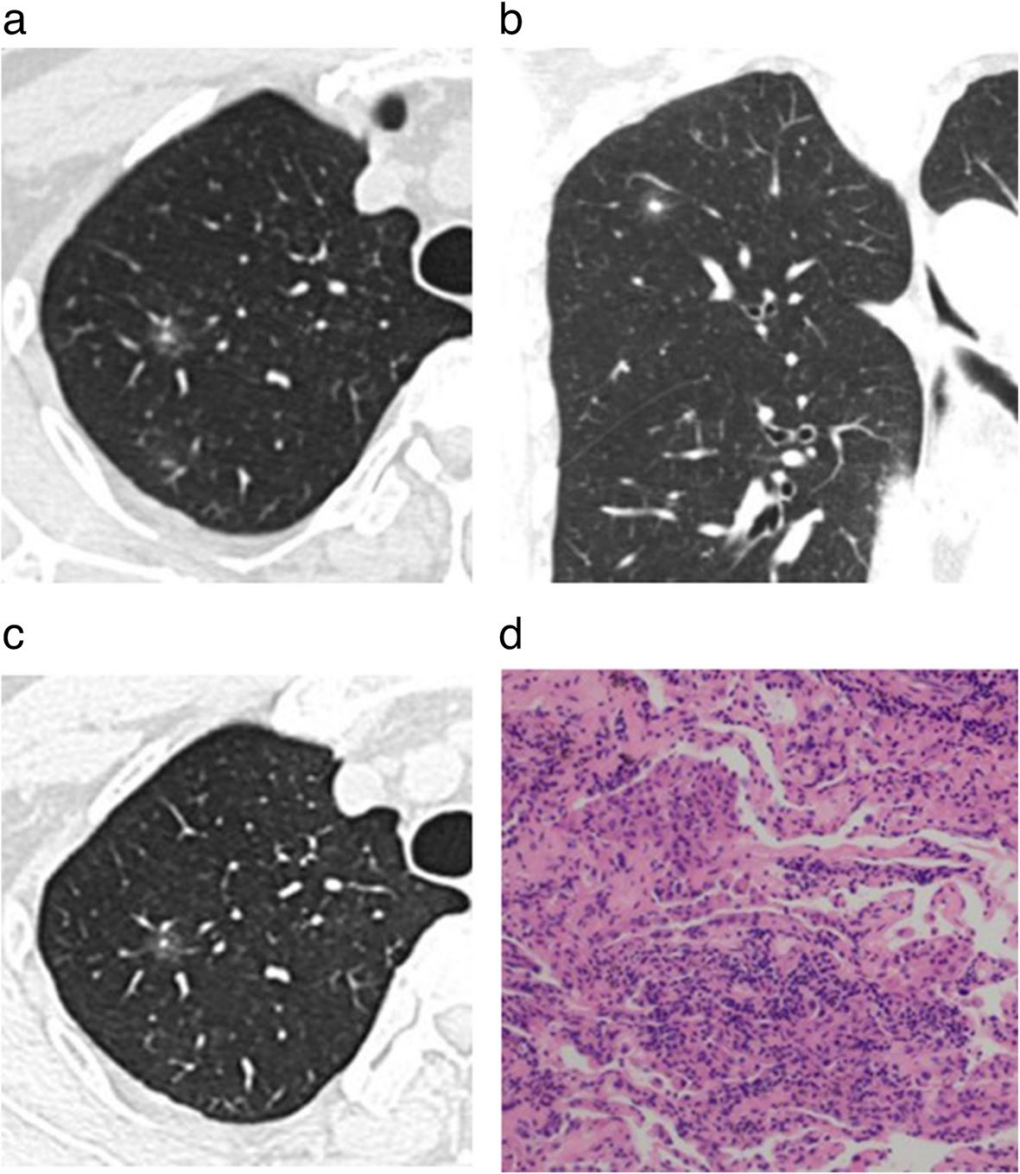
Nonabsorbable benign pulmonary subsolid nodule in a 60-year-old man. **a**, **b** The initial axial and coronal lung window CT images showed a subsolid nodule with a well-defined margin and a target sign in the right upper lobe. **c** Three months later, the axial lung window CT image showed nodule persistence. **d** Photomicrography (hematoxylin and eosin staining; magnification × 200) confirmed the presence of an inflammatory nodule

**Fig. 4:**
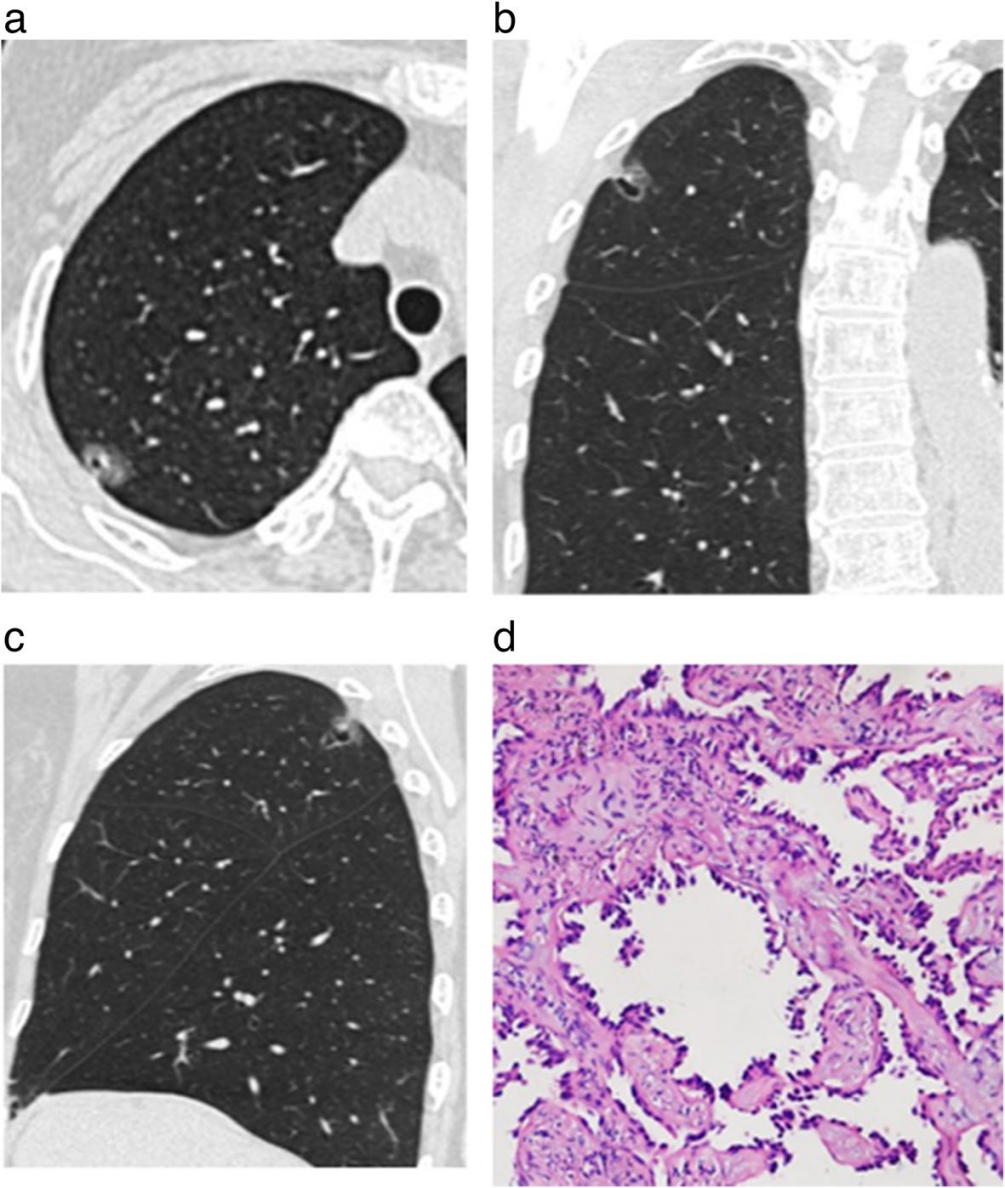
Malignant pulmonary subsolid nodule in a 72-year-old woman. **a**–**c** The initial axial, coronal, and sagittal lung window CT images showed a subsolid nodule with a well-defined margin and bubble lucency in the right upper lobe. **d** Photomicrography (hematoxylin and eosin staining; magnification × 100) confirmed invasive adenocarcinoma

The non-contrast CT features of AB-SSNs, NB-SSNs, and M-SSNs are displayed in Table 3. NB-SSNs were significantly smaller than M-SSNs (*p* < 0.017). Ill-defined margins were more frequent in AB-SSNs than in NB-SSNs and M-SSNs (all *p* < 0.017). Irregular shape, target sign, and lower CT value of GGO areas were more common in both AB-SSNs and NB-SSNs than in M-SSNs, whereas bubble lucency was more common in M-SSNs than in both AB-SSNs and NB-SSNs (all *p* < 0.017). Furthermore, thickened interlobular septa and satellite lesions were more common in AB-SSNs than in M-SSNs, whereas pleural retraction was more frequent in M-SSNs than in AB-SSNs (all *p* < 0.017) (Figs. 2, 3 and 4). However, no significant between-group differences were observed in the location or density of nodules, air bronchogram, abnormal intranodular vessel sign, or pleural attachment (all *p* > 0.017).

**Table 3:** Comparison of noncontrast computed tomography features among absorbable benign, nonabsorbable benign, and malignant subsolid nodules

Characteristics	AB-SSNs (*n* = 69)	NB-SSNs (*n* = 70)	M-SSNs (*n* = 297)	*p*_1_*	*p*_2_*	*p*_3_*
Location				0.949 ^a^	0.214 ^a^	0.244 ^a^
Upper lobe	44 (63.77%)	45 (64.29%)	212 (71.38%)			
Middle/lower lobe	25 (36.23%)	25 (35.71%)	85 (28.62%)			
Size (mm)				0.123 ^b^	0.019 ^b^	** < 0.001** ^**b**^
Average	10.00 ± 6.00	9.00 ± 5.00	11.00 ± 4.00			
Range	5–23	5–21	8–27			
Margin				** < 0.001** ^**a**^	** < 0.001** ^**a**^	0.661 ^a^
Well-defined	9 (13.04%)	39 (55.71%)	174 (58.59%)			
Ill-defined	60 (86.96%)	31 (44.29%)	123 (41.41%)			
Shape				0.556 ^a^	** < 0.001** ^**a**^	** < 0.001**^**a**^
Regular	39 (56.52%)	43 (61.43%)	243 (81.82%)			
Irregular	30 (43.48%)	27 (38.57%)	54 (18.18%)			
Density				0.554 ^a^	0.566 ^a^	0.183 ^a^
GGO	34 (49.28%)	38 (54.29%)	135 (45.45%)			
PSO	35 (50.72%)	32 (45.71%)	162 (54.55%)			
CT value of GGO areas (HU)	− 633.04 ± 117.31	− 635.53 ± 112.98	− 592.00 ± 167.00	0.899 ^c^	**0.001** ^**b**^	** < 0.001**^**b**^
Target sign	12 (17.39%)	9 (12.86%)	11 (3.70%)	0.455 ^a^	** < 0.001** ^**a**^	**0.006** ^**a**^
Air bronchogram	4 (5.80%)	4 (5.71%)	33 (11.11%)	1.000 ^a^	0.187 ^a^	0.177 ^a^
Bubble lucency	0 (0%)	2 (2.86%)	44 (14.81%)	0.496 ^a^	**0.001** ^**a**^	**0.007** ^**a**^
Abnormal intranodular vessel sign	7 (10.14%)	9 (12.86%)	46 (15.49%)	0.616 ^a^	0.256 ^a^	0.579 ^a^
Thickened interlobular septa	6 (8.70%)	1 (1.43%)	3 (1.01%)	0.116 ^a^	**0.001** ^**a**^	0.573 ^a^
Satellite lesions	9 (13.04%)	4 (5.71%)	11 (3.70%)	0.138 ^a^	**0.005** ^**a**^	0.668 ^a^
Pleural retraction	2 (2.90%)	7 (10.00%)	48 (16.16%)	0.175 ^a^	**0.004** ^**a**^	0.194 ^a^
Pleural attachment	13 (18.84%)	13 (18.57%)	65 (21.89%)	0.968 ^a^	0.578 ^a^	0.542 ^a^

*AB-SSNs* Absorbable benign subsolid nodules, *NB-SSNs* Nonabsorbable benign subsolid nodules, *M-SSNs* Malignant subsolid nodules, *GGO* Ground glass opacity, *PSO* Part-solid opacity, *CT* Computed tomography

^*^Bonferroni correction in which *p* < 0.017 is significant. *p*_1_: the *p*-value of AB-SSNs vs. NB-SSNs; *p*_2_: the *p-*value of AB-SSNs vs. M-SSNs; *p*_3_: the *p*-value of NB-SSNs vs. M-SSNs

^a^Chi-squared test

^b^Mann–Whitney *U* test

^c^Two independent-sample Student’s *t*-tests

### Development of multivariate logistic regression models

Three models were built by incorporating clinical and non-contrast CT features to distinguish different SSN types. Model 1 was used to differentiate AB-SSNs and NB-SSNs. Compared with NB-SSNs, detected during follow-up CT and ill-defined margins were independent predictors of AB-SSNs, with an AUC of 0.748 (95% CI, 0.667–0.830; *p* < 0.001) and accuracy of 71.2% (Figure S1a). Model 2 was used to differentiate AB-SSNs and M-SSNs. Compared with M-SSNs, younger age, the presence of respiratory symptoms, detected during follow-up CT, ill-defined margin, irregular shape, target sign, lower GGO CT values, thickened interlobular septa, and the absence of bubble lucency were independent predictors of AB-SSNs, with an AUC of 0.920 (95% CI, 0.881–0.959; *p* < 0.001) and accuracy of 90.4% (Figure S1b). Model 3 was used to differentiate NB-SSNs and M-SSNs. Compared with M-SSNs, detected during follow-up CT, smaller nodule size, irregular shape, target sign, and lower GGO CT values were independent predictors of NB-SSNs, with an AUC of 0.824 (95% CI, 0.767–0.881; *p* < 0.001) and accuracy of 85.3% (Figure S1c).

### External validation of multivariate logistic regression models

The external validation cohort comprised 151 patients (mean age: 53 ± 13 [range, 18–82] years, 53 men and 98 women) from another center, including 35 patients with AB-SSNs (mean age: 52 ± 16 [range, 18–82] years, 20 men and 15 women), 30 patients with NB-SSNs (mean age: 55 ± 12 [range, 20–78] years, 11 men and 19 women), and 86 patients with M-SSNs (mean age: 53 ± 12 [range, 30–71] years, 22 men and 64 women). Table 4 illustrates the clinical and imaging characteristics used for external validation of the three aforementioned models. The external validation cohort obtained an AUC of 0.790 (95% CI, 0.679–0.900;* p* < 0.001) and accuracy of 73.9% for model 1 (Figure S1d), an AUC of 0.912 (95% CI, 0.859–0.966; *p* < 0.001) and accuracy of 81.8% for model 2 (Figure S1e), and an AUC of 0.794 (95% CI, 0.693–0.895; *p* < 0.001) and accuracy of 80.2% for model 3 (Figure S1f).

**Table 4:** The clinical and imaging characteristics used for the external validation of three regression models

**External validation of model 1 (AB-SSNs vs. NB-SSNs)**		
**Characteristics**	**AB-SSNs (*n* = 35)**	**NB-SSNs (*n* = 30)**
Detected time		
Initial CT	25 (71.43%)	28 (93.33%)
Follow-up CT	10 (28.57%)	2 (6.67%)
Margin		
Well-defined	5 (14.29%)	18 (60.00%)
Ill-defined	30 (85.71%)	12 (40.00%)
**External validation of model 2 (AB-SSNs vs. M-SSNs)**		
**Characteristics**	**AB-SSNs (*n* = 35)**	**M-SSNs (*n* = 86)**
Age (years)		
Average	52 ± 16	53 ± 12
Range	18–82	30–71
Respiratory symptoms		
Presence	4 (11.43%)	10 (11.63%)
Absence	31 (88.57%)	76 (88.37%)
Detected time		
Initial CT	25 (71.43%)	86 (100%)
Follow-up CT	10 (28.57%)	0 (0%)
Margin		
Well-defined	5 (14.29%)	76 (88.37%)
Ill-defined	30 (85.71%)	10 (11.63%)
Shape		
Regular	32 (91.43%)	68 (79.07%)
Irregular	3 (8.57%)	18 (20.93%)
Target sign		
Presence	6 (17.14%)	4 (4.65%)
Absence	29 (82.86%)	82 (95.35%)
CT value of GGO areas (HU)	− 722.00 ± 171.00	− 606.22 ± 106.02
Thickened interlobular septa		
Presence	2 (5.71%)	2 (2.33%)
Absence	33 (94.29%)	84 (97.67%)
Bubble lucency		
Presence	0 (0%)	18 (20.93%)
Absence	35 (100%)	68 (79.07%)
**External validation of model 3 (NB-SSNs vs. M-SSNs)**		
**Characteristics**	**NB-SSNs (*n* = 30)**	**M-SSNs (*n* = 86)**
Detected time		
Initial CT	28 (93.33%)	86 (100%)
Follow-up CT	2 (6.67%)	0 (0%)
Size (mm)		
Average	8.00 ± 3.00	10.00 ± 3.00
Range	5–14	5–19
Shape		
Regular	19 (63.33%)	68 (79.07%)
Irregular	11 (36.67%)	18 (20.93%)
Target sign		
Presence	6 (20.00%)	4 (4.65%)
Absence	24 (80.00%)	82 (95.35%)
CT value of GGO areas (HU)	− 661.50 ± 98.00	− 606.22 ± 106.02

*AB-SSNs* Absorbable benign subsolid nodules, *NB-SSNs* Nonabsorbable benign subsolid nodules, *M-SSNs* Malignant subsolid nodules, *CT* Computed tomography, *GGO* Ground glass opacity

### Development trends for benign SSNs

**Fig. 5:**
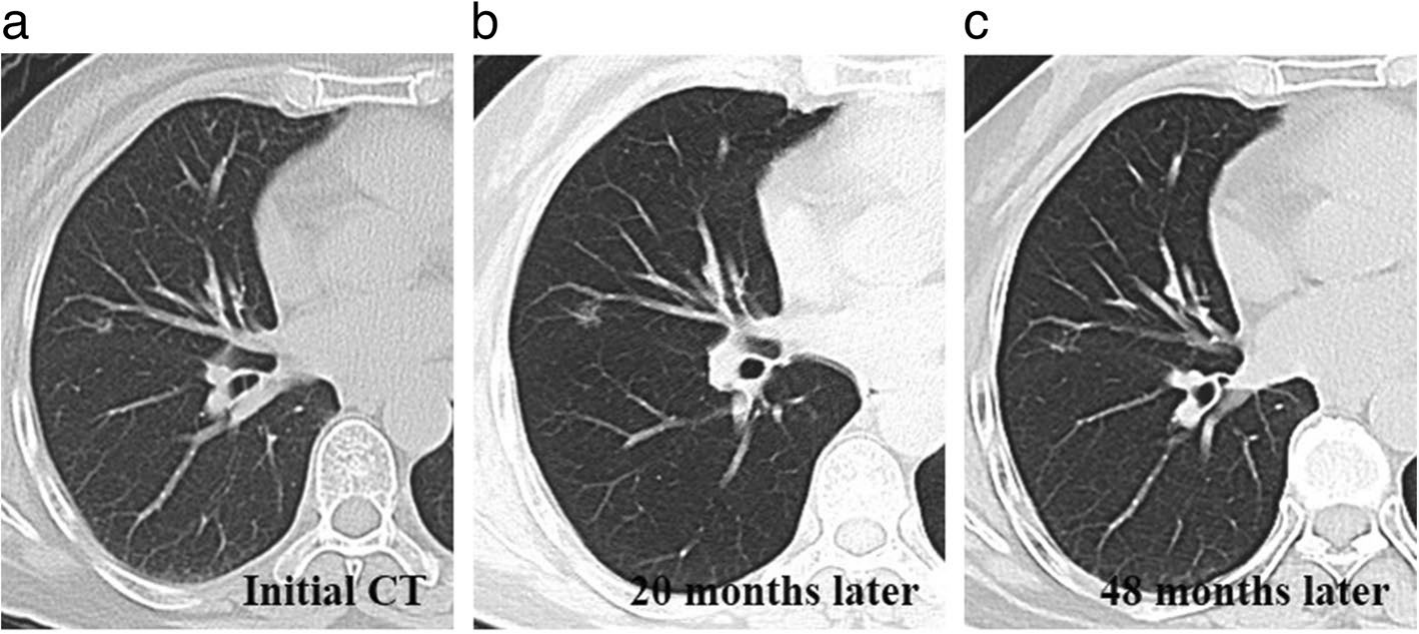
Morphological evolution of absorbable benign pulmonary subsolid nodule during follow-up CT in a 57-year-old woman. **a** The initial lung window CT image showed a subsolid nodule in the right middle lobe. **b** Twenty months later, the lung window CT image showed that the nodule had increased in size. **c** Forty-eight months later, the lung window CT image showed absorption of the nodule

**Fig. 6:**
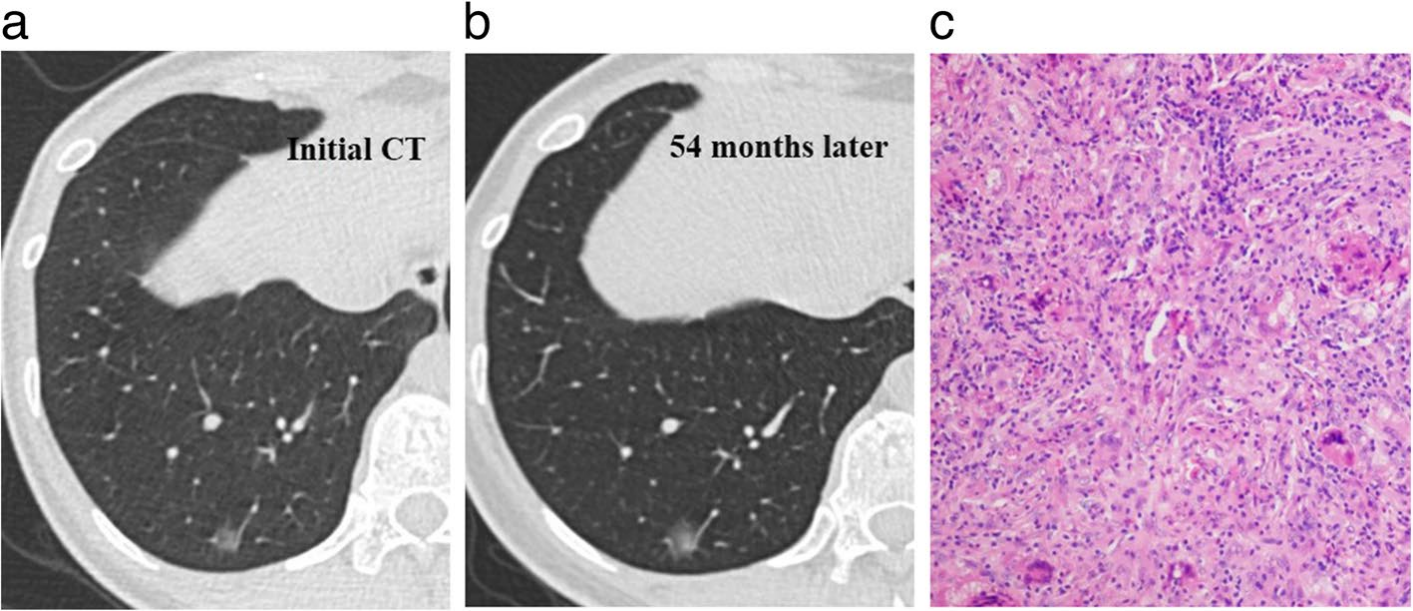
Morphological evolution of a nonabsorbable benign pulmonary subsolid nodule on follow-up CT in a 34-year-old woman. **a** The initial lung window CT image showed a subsolid nodule in the right lower lobe. **b** Fifty-four months later, the lung window CT image showed that the nodule had increased in size and density. **c** Photomicrography (hematoxylin and eosin staining; magnification × 200) confirmed granuloma with inflammatory cell infiltration

Among the 69 AB-SSNs, 68 nodules (98.55%) gradually decreased in size and had disappeared at the time of follow-up CT. One nodule (1.45%) progressed and then gradually resolved (Fig. 5). This nodule was 5 mm in diameter on the initial CT scan, then showed slight enlargement after 20 months, and subsequently underwent noticeable absorption after another 28 months. Among the 70 NB-SSNs, 67 nodules (95.71%) had no obvious changes on follow-up CT. The interval between the initial CT and the preoperative CT ranged from 90 to 1404 days with a mean of 95 (± 85) days. Three nodules (4.29%) had progressed on the follow-up CT. The intervals between the initial CT and preoperative CT in these three patients were 96, 159, and 1660 days. Of these, one nodule was 8 mm in diameter on the initial CT and had increased to 12 mm after 54 months. This nodule was surgically confirmed to have a granuloma with inflammatory cell infiltration (Fig. 6). The other two nodules enlarged within a short time after the first CT scan (3 and 5 months) and were surgically confirmed to be inflammatory.

## Discussion

Familiarity with the clinical and imaging findings of SSNs can help clinicians select optimal therapeutic strategies, minimize overtreatment of benign nodules, and relieve patients’ physical and economic burdens. In this study, the clinical and non-contrast CT features of 69 patients with AB-SSNs, 70 with NB-SSNs, and 297 with M-SSNs were compared and three regression models for their differentiation were developed. The development trends of benign SSNs were also analyzed.

Our finding that patients with AB-SSNs were younger and more likely to have respiratory symptoms than those with M-SSNs was consistent with prior studies [15–17]. Interestingly, we found that newly observed nodules, which were absent on the initial CT and detected during follow-up CT, were most common in AB-SSNs, followed by NB-SSNs, suggesting that SSNs observed during follow-up are strongly indicative of benignity, especially absorbable benignity, whereas M-SSNs tend to persist for longer. This finding could be attributable to the pathological differences between inflammatory and M-SSNs. Generally, AB-SSNs show inflammatory exudate or hemorrhage in the alveolar space; NB-SSNs are associated with hyperplasia of granulation tissue, fibrosis, and infiltration of chronic inflammatory cells; and M-SSNs are associated with the invasion of tumor cells along preexisting alveolar structures without completely occupying the alveolar space [18–21]. Therefore, attention to the detection time of SSNs on follow-up CT may help in differentiating between benign and malignant pulmonary nodules. Furthermore, less aggressive follow-up and management may be appropriate for newly observed SSNs.

Consistent with previous research, we found NB-SSNs to be smaller than M-SSNs [9]. We also found that ill-defined nodule margins were more common in AB-SSNs than in M-SSNs and NB-SSNs. Moreover, irregular shapes and lower CT values of GGO areas were more common in benign SSNs than in M-SSNs, which is consistent with previous findings [8, 10, 12, 13, 22]. The distinct pathologies mentioned above may explain these results. The target sign also appears to be indicative of benign SSNs, which may correlate to granuloma formation in the center, mild fibrous tissue hyperplasia of the interstitium, inflammatory cell infiltration, and/or alveoli exudate in the periphery [9]. In line with previous studies, we found bubble lucency to be highly indicative of malignancy [10, 12, 22, 23]. In addition, thickened interlobular septa and satellite lesions were more common in AB-SSNs than in M-SSNs. This may be because acute inflammation in AB-SSNs often involves adjacent interstitium or alveoli [22, 24]. Several studies have shown pleural retraction to be common in M-SSNs [11, 12], suggesting it to be an indicator for malignancy [22, 25, 26], and our findings supported this.

In addition, three regression models for differentiating SSNs obtained fair-to-excellent diagnostic performances in both training and external validation cohorts. The most effective factors associated with benign SSNs in each model were also identified. Familiarity with these features may be advantageous to the accurate diagnosis and early treatment of SSNs.

A thorough understanding of the dynamic development of pulmonary nodules on follow-up CT plays a key role in identifying their nature. In this study, we observed unusual behavior in four benign SSNs. The first of these was an AB-SSN, which was detected on initial CT, was enlarged after 20 months, and ultimately resolved after another 28 months. Based on preexisting chronic inflammation, we speculated that this may have resulted from a secondary infection. Such a change may cause increased production and activation of macrophages in the lesion, which can phagocytose necrotic tissue fragments and pathogens and trigger the secretion of various bioactive substances and enzymes to promote the absorption of fibrous tissue in the nodule. The second unexpected progression was an NB-SSN, which exhibited an increase in size and density 54 months after the initial CT and was surgically confirmed as granulomas with inflammatory cell infiltration. This phenomenon could again be attributed to acute infection, but in this case, it was secondary to a preexisting granuloma. The other two progressions were also NB-SSNs. Both of these enlarged over a short time after the initial CT and were surgically confirmed as inflammatory nodules. In inflammatory lesions, early pathological changes often involve a small amount of inflammatory exudate in the alveolar cavity, followed by chronic inflammatory cell infiltration and fibrous tissue proliferation. Correspondingly, the nodule may present as an SSN in the early stages and then show increased size and density on follow-up CT.

This study had several limitations. First, there may have been selection bias due to the retrospective design. Second, given that it is very difficult to obtain pathological specimens after each follow-up CT scan, the pathological basis for the unusual behavior observed in four benign nodules is currently unknown and we can only speculate on the possible mechanism. Third, as postcontrast imaging data on nodules were not available in this study, we mainly focused on the non-contrast CT features of SSNs. Further correlational studies with larger samples are warranted.

## Conclusions

In conclusion, our findings demonstrate that AB-SSNs, NB-SSNs, and M-SSNs have different clinical and imaging characteristics. Significantly, a miniscule number of benign SSNs can also progress to resemble malignancy. A thorough understanding of the clinical and imaging features and development trends of benign SSNs may contribute to their early diagnosis and better management.

### Supplementary Information

**Additional file 1: Figure S1.** The receiver operator characteristic (ROC) curves of three regression models in the training and external validation cohorts.

## Data Availability

The datasets used and/or analyzed during the current study are available from the corresponding author upon reasonable request.

## Abbreviations

AB-SSNs: Absorbable benign subsolid nodules
AUC: Area under the curve
GGO: Ground-glass opacity
ICC: Intraclass correlation coefficient
M-SSNs: Malignant subsolid nodules
NB-SSNs: Nonabsorbable benign subsolid nodules
PSO: Part-solid opacity
ROI: Region of interest
SSNs: Subsolid nodules
