# Supplementary material for: Clinical and non-contrast computed tomography characteristics and disease development in patients with benign pulmonary subsolid nodules with a solid component ≤ 5 mm
Source: Insights Imaging. 2024 Jan 8;15:6. doi: 10.1186/s13244-023-01585-5 (PMC10774240; doi:10.1186/s13244-023-01585-5)
Supplement: Supplementary file 1 — Additional file 1: Figure S1. The receiver operator characteristic (ROC) curves of three regression models in the training and external validation cohorts. [file 13244_2023_1585_MOESM1_ESM.pdf]

Clinical and non-contrast computed tomography characteristics and disease development in patients with benign pulmonary subsolid nodules with a solid component ≤5 mm

ELECTRONIC SUPPLEMENTARY MATERIAL

Figure S1. The receiver operator characteristic (ROC) curves of three regression models in the training and external validation cohorts.

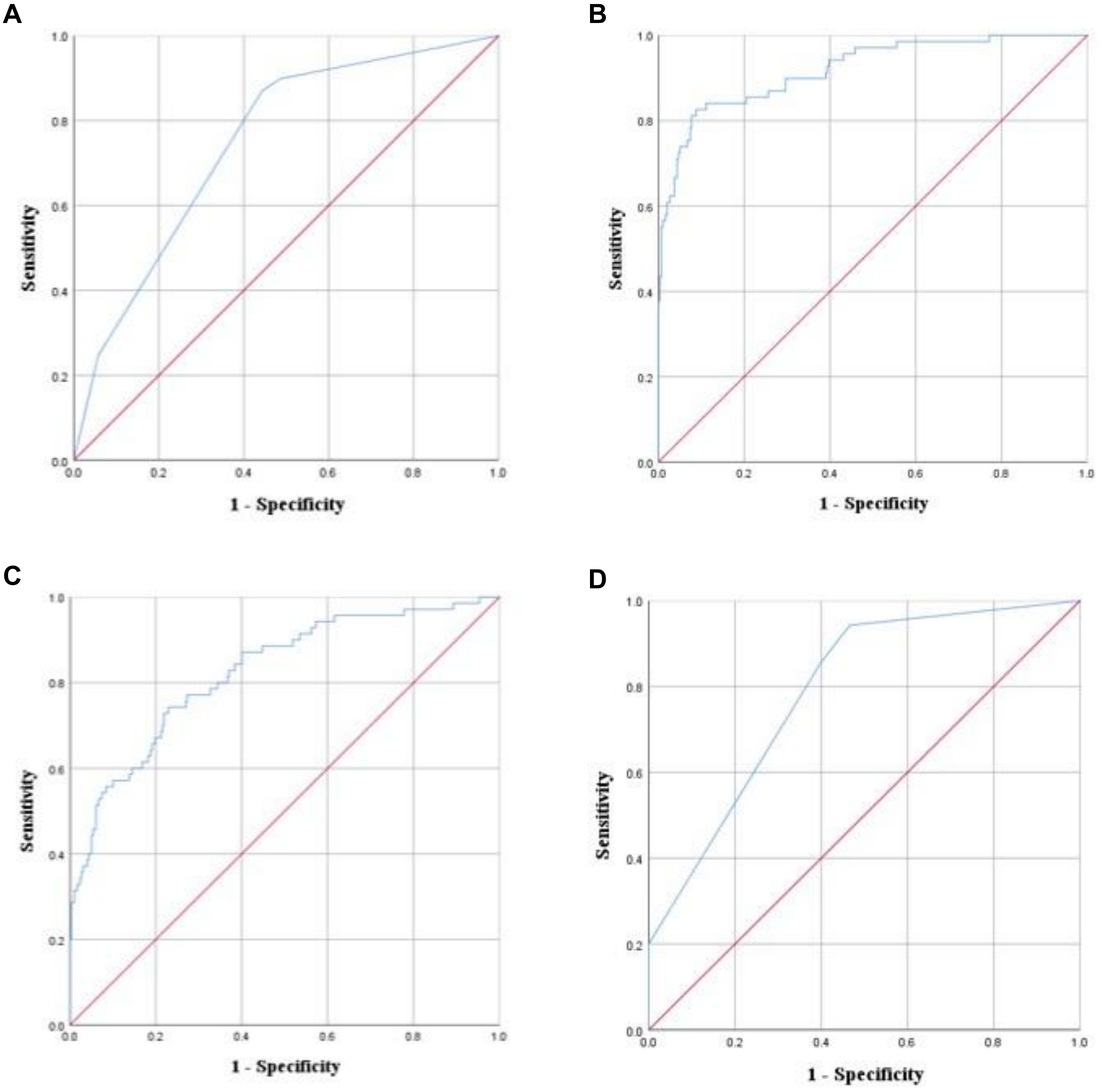

E

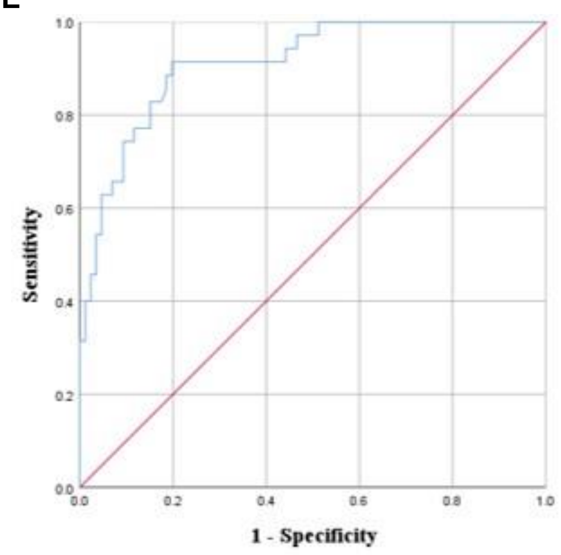

F

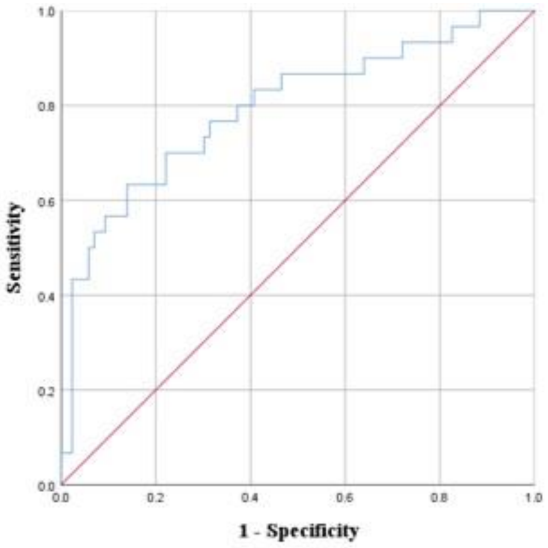

A. The ROC curve of model 1 (AB-SSNs vs. NB-SSNs) in the training cohort.

B. The ROC curve of model 2 (AB-SSNs vs. M-SSNs) in the training cohort.

C. The ROC curve of model 3 (NB-SSNs vs. M-SSNs) in the training cohort.

D. The ROC curve of model 1 (AB-SSNs vs. NB-SSNs) in the external validation cohort.

E. The ROC curve of model 2 (AB-SSNs vs. M-SSNs) in the external validation cohort.

F. The ROC curve of model 3 (NB-SSNs vs. M-SSNs) in the external validation cohort.
